# Complete lung collapse in a young adolescent

**DOI:** 10.7196/SARJ.2018.v24i2.209

**Published:** 2018-06-21

**Authors:** T H Ahmed, A Esmail, G Calligaro, K Dheda

**Affiliations:** Division of Pulmonology, Department of Medicine, University of Cape Town Lung Institute and Groote Schuur Hospital, Cape Town, South Africa

**Keywords:** carcinoid, bronchial, neuroendocrine tumor, typical carcinoid, atypical carcinoid

## Abstract

Bronchial carcinoid tumours (BCTs) arise from the neuroendocrine cells of the bronchial epithelium known as Kulchitsky cells. They represent ~25% of all carcinoid tumours, usually have a central distribution, and present with features of bronchial obstruction. They are the most common lung malignancy in children. Here we report the case of a 14-year-old girlwith chronic respiratory symptoms and left lung collapse due to bronchial carcinoid. The differential diagnosis of segmental, lobar or total lung collapse in a young person also includes mucus plugging or foreign body aspiration.

## Background

Bronchial carcinoid tumours (BCTs) were first described in 1888 by Lubarsch, who found multiple tumours in the distal ileum of two patients on autopsy. In 1907, Oberndorfer coined the term ‘*karzinoid tumoren*’ to describe ileal tumours that appeared to behave less aggressively than typical adenocarcinomas.^[1]^ BCTs represent ~25% of all carcinoid tumours – the remainder can be seen primarily in the gastrointestinal tract, but also the mediastinum, thymus, liver, pancreas, ovaries, prostate and kidneys. BCTs are divided into typical and atypical tumours based on their pathological tumour grade. Typical BCTs are slow-growing tumours that rarely metastasise, whereas atypical tumours metastasise early to the hilar or mediastinal nodes and are associated with a higher recurrence rate.^[2]^ Surgery is the gold standard for patients with resectable lung carcinoids, but treatment options are very limited for patients with metastatic or unresectable disease. The incidence of BCTs has increased over the past 30 years, which may be due to a greater effort to better characterise these neoplasms, with the help of a multidisciplinary approach.

## Case

**Fig. 1:**
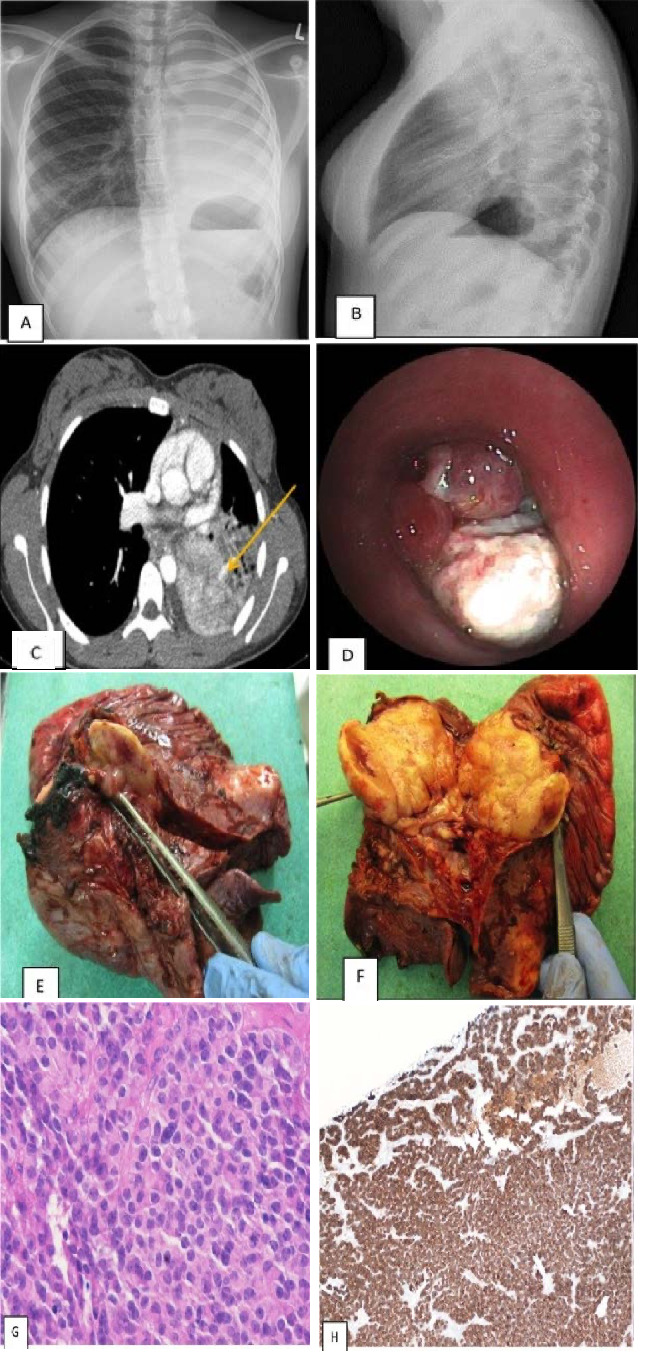
(A and B) Chest radiograph PA and lateral showing left lung collapse (left hemithorax homogenous opacity with volume loss). (C) Mediastinal window shows a well-defined intraluminal growth in the the left main bronchus occluding the airway (arrow). (D) Fibre optic bronchoscopy, reddish-yellow polypoidal mass occluding whole lumen of the left main bronchus. (E and F) Post pneumonectomy view. Huge bulky tumour removed from left main bronchus. (G) Round nuclei and ample amounts of granular cytoplasm seen at high power. (H) Stain positive for chromogranin and synaptophysin.

A 14-year-old girl was referred to our unit from the emergency department at our hospital with a short history of dyspnoea and left-sided chest pain. Her symptoms worsened acutely following a suspected lower respiratory tract infection. She had a three-month history of progressive exercise limitation and wheeze. She was presumptively diagnosed with asthma and started on bronchodilators. Clinical examination revealed tachypnoea and saturations of 92% on room air, reduced expansion of the left chest, tracheal deviation to the left, and absent breath sounds over the left hemithorax. A chest X-ray showed complete collapse of the left lung with cut-off in the proximal left main bronchus (Figs 1A and 1B). There was no history of foreign body aspiration. A flexible bronchoscopy was done which showed a smooth, reddish-yellow polypoidal mass occluding the entire lumen of the left main bronchus (Fig. 1C). Due to the proximity of the mass to the carina, and its vascularity, endobronchial biopsies were not performed. Computed tomography (CT) of the chest showed the endobronchial mass to be part of a large inhomogeneous lesion occupying the entire left upper lobe (Fig. 1D), and the patient was referred for pneumonectomy. The surgical specimen is shown in Figs 1E and 1F. Histological evaluation showed the presence of uniform polygonal cells with finely granular chromatin in round nuclei and a moderate amount of eosinophilic cytoplasm without any nuclear atypia, mitosis or necrosis (Fig. 1G). Immunohistochemistry showed neuroendocrine differentiation of tumour cells with cytoplasmic positivity of cytokeratin, chromogranin A and synaptophysin (Fig. 1H). A diagnosis of typical carcinoid tumour was confirmed.

## Discussion

BCTs are rare neuroendocrine neoplasms of the lung, which is the second most common site of carcinoid tumours after the abdomen. BCTs may be central or peripheral, and have been reported in all age groups, but the peak incidence is around the fourth to fifth decade of life.^[3]^ About 80% of bronchial carcinoids arise centrally in the main, lobar and segmental bronchi with no specific lobar distribution.^[4]^ Central tumours are usually symptomatic with features of bronchial obstruction, whereas peripheral tumours are usually asymptomatic.^[4]^ The most common symptoms are haemoptysis, cough, recurrent chest infections, fever, unilateral wheezing and dyspnoea.^[5]^

Due to the high tumour vascularity, haemoptysis occurs in at least 50% of patients,^[6]^ whereas 25% of patients are diagnosed incidentally.^[7]^ Patients are often misdiagnosed with airways disease, as occurred with our patient. Diagnosis in our case was further delayed as this patient had no haemoptysis despite having a centrally located tumour.

Aetiologically, no association has been found between BCTs and cigarette smoking, ambient radiation or exposure to other known carcinogens;^[8]^ however, a recent study described a possible association between atypical carcinoids and smoking.^[9]^

Radiologically, typical and atypical BCTs have similar features, depending on tumour location. Most BCTs appear on chest radiographs or chest CT as circumscribed, centrally located lesions with a diameter of 2 - 5 cm.^[10]^

They are therefore usually easily accessible via flexible bronchoscopy. Due to their high vascularity, there has been a historical concern about the safety of endobronchial biopsy in BCTs; however, recent studies, as well as the British Thoracic Society, refer to the incidence of serious bleeding complications during bronchoscopic biopsy as being very low (<1%).^[11]^ Final diagnosis is based on histology and confirmed with immunohistochemical staining for neuroendocrine markers.

Resection is the treatment of choice,^[12]^ and surgical procedures include pneumonectomy, lobectomy, segmentectomy, sleeve resection and wedge resection. The aim is to remove the primary tumour and affected lymph nodes radically, saving as much of the lung parenchyma as possible. Our patient presented with complete obstruction of the left lung with a mass occupying a significant proportion of the lung parenchyma, and, as a result, underwent pneumonectomy. The survival following surgery for BCTs is excellent: a 5-year survival rate of 94% was observed in 1 109 patients with typical BCTs by the European Society of Thoracic Surgeons Neuroendocrine Tumours Working Group, and a 3-year survival rate of 67% was observed in the USA in a database analysis of 441 patients^[13,14]^ following resection of the tumour.

Chemotherapy and radiation therapy is largely unhelpful if unresectable or metastatic disease is present. Typical bronchial carcinoids generally have an excellent prognosis, whereas atypical bronchial carcinoids have a poorer prognosis.

Delay in diagnosis can result in potential complications from increased tumour size causing obstruction of the bronchus and destructive changes in the lung. Our patient had an uneventful recovery, with no residual respiratory symptoms or functional limitation.

## References

[1] Oberndorfer S (1907). Karzinoide tumoren des dunndarms.. Frankfurt Z Pathol.

[2] Gonzalez JM, Garcia-Yuste M, Moreno-Mata N (2005). Typical and atypical carcinoid tumors (NEC grades 1 and 2): Prognostic factors in metastases and local recurrence.. Lung Cancer.

[3] Tsilimigras DI, Moris D, Ntanasis-Stathopoulos I, Patrini D, Panagiotopoulos N (2017). Endobronchial carcinoid tumor totally occluding the left main bronchus without producing symptoms of bronchial obstruction.. In Vivo.

[4] Nessi R, Basso PR, Basso SR, Bosco M, Blanc M, Uslenghi C (1991). Bronchial carcinoid tumors: Radiologic observations in 49 cases.. J Thorac Imaging.

[5] Zuetenhorst JM, Taal BG (2005). Metastatic carcinoid tumors: A clinical review.. Oncologist.

[6] Dusmet ME, McKneally MF (1996). Pulmonary and thymic carcinoid tumors.. World J Surg.

[7] Ducrocq X, Thomas P, Massard G (1998). Operative risk and prognostic factors of typical bronchial carcinoid tumors.. The Annals of thoracic surgery.

[8] Davila DG, Dunn WF, Tazelaar HD, Pairolero PC (1993). Bronchial carcinoid tumors.. Mayo Clin Proc.

[9] Fink G, Krelbaum T, Yellin A (2001). Pulmonary carcinoid: Presentation, diagnosis, and outcome in 142 cases in Israel and review of 640 cases from the literature.. Chest.

[10] Hage R, de la Rivière AB, Seldenrijk CA, Van den Bosch JM (2003). Update in pulmonary carcinoid tumors: A review article.. Ann Surg Oncol.

[11] Kaifi JT, Kayser G, Ruf J, Passlick B (2015). The diagnosis and treatment of bronchopulmonary carcinoid.. Deutsches Ärzteblatt International.

[12] Caplin ME, Baudin E, Ferolla P (2015). Pulmonary neuroendocrine (carcinoid) tumors: European Neuroendocrine Tumor Society expert consensus and recommendations for best practice for typical and atypical pulmonary carcinoids.. Ann Oncol.

[13] Filosso PL, Guerrera F, Evangelista A (2015). Prognostic model of survival for typical bronchial carcinoid tumours: analysis of 1109 patients on behalf of the European Association of Thoracic Surgeons (ESTS) Neuroendocrine Tumours Working Group.. Eur J Cardio-Thorac Surg.

[14] Steuer CE, Behera M, Kim S (2015). Atypical carcinoid tumor of the lung: A surveillance, epidemiology, and end results database analysis.. Thorac Oncol.

